# Effect of Probiotic *Lacticaseibacillus rhamnosus* LB1.5 on Anxiety-like Behavior, Neuroprotection and Neuroinflammation Markers of Male Mice Fed a High-Fat Diet

**DOI:** 10.3390/nu16060879

**Published:** 2024-03-18

**Authors:** Natália Perin Schmidt, Patrícia Molz, Brenda Santos Fraga, Nicole Hiller Bondarczuk, Priscila Dutra Silveira, Milena Henrique Ferri, Thais Busatto Crestani, Gabriela Merker Breyer, Giuliano Rizzoto Guimarães, Amanda de Souza da Motta, Renata Padilha Guedes, Márcia Giovenardi

**Affiliations:** 1 Graduate Program in Biosciences, Federal University of Health Sciences of Porto Alegre (UFCSPA), Porto Alegre 90050-170, Brazil; nataliaps@ufcspa.edu.br (N.P.S.); patricia.molz@gmail.com (P.M.); brenda.fraga@ufcspa.edu.br (B.S.F.); nicoleh@ufcspa.edu.br (N.H.B.); priscilasilveira@ufcspa.edu.br (P.D.S.); milena.ferri@ufcspa.edu.br (M.H.F.); renata.guedes@ufcspa.edu.br (R.P.G.); 2 Biomedicine Course, Federal University of Health Sciences of Porto Alegre (UFCSPA), Porto Alegre 90050-170, Brazil; thais.crestani@ufcspa.edu.br; 3 Graduate Program in Agricultural Microbiology and Environment, Federal University of Rio Grande do Sul (UFRGS), Porto Alegre 90050-170, Brazil; gabibreyer@hotmail.com (G.M.B.); amanda.motta@ufrgs.br (A.d.S.d.M.); 4 Pathology Laboratory, Federal University of Health Sciences of Porto Alegre (UFCSPA), Porto Alegre 90050-170, Brazil; giuliano@ufcspa.edu.br

**Keywords:** DOHaD, Nrf2, Sirt1, Bdnf, anxiety-like behavior

## Abstract

Probiotic supplementation has been identified as a potential target to reduce inflammatory mediators associated with obesity. Therefore, this study assessed the effect of probiotic *Lacticaseibacillus rhamnosus* LB1.5 on anxiety-like behavior, gene expression in the prefrontal cortex, and neuroinflammation in the cerebral cortex and hippocampus of male mice fed a high-fat diet. Mice aged 21 days were divided into four groups: control (CONT), control plus probiotic (CONT + PROB), high-fat diet (HFD), and high-fat diet plus probiotic (HFD + PROB), and fed for 13 weeks. The probiotic *Lact. rhamnosus* 1.5 (3.1 × 108 CFU/mL, derived from raw buffalo milk) was administered by gavage three times a week. Probiotic supplementation provided an anxiolytic effect in CONT and HFD. The IL-6 showed lower levels after probiotic supplementation in the HFD. Regarding immunoreactivity for GFAP in the cerebral cortex, we demonstrated that animals HFD-fed had a reduction in cells number compared to CONT. In the hippocampus, we found an interaction between diet and supplementation, as well as an effect of probiotic supplementation. A higher number of Th positive cells was observed in the cerebral cortex in mice fed HFD. *Lact. rhamnosus* LB1.5 supplementation decreased serum IL-6 levels in HFD-fed mice and promoted a reduction in anxiety-like behavior.

## 1. Introduction

The relationship between the consumption of a high-fat diet (HFD) and the development of overweight and obesity is already well-established in the scientific literature, both in studies with animal models [1,2,3,4] and in humans [5].

Furthermore, as studies have been developed in the branch of science known as the developmental origins of health and disease (DOHaD), associations have been demonstrated between conditions occurring in the early stages of somatic development and the increase in the risk for chronic diseases throughout life, such as obesity, diabetes and cardiovascular diseases [6,7]. Although some research indicates advantages of pre- and probiotics for pregnant and breastfeeding mothers, the enduring impact of these supplements on the neurological and immunological growth of individuals remains uncertain [7].

Consequently, an increasing focus has been placed on non-pharmacological strategies to prevent or mitigate the negative outcomes observed in obese individuals. Among these strategies, the supplementation of probiotics has gained attention [8,9]. As defined by the World Health Organization, probiotics are live microorganisms that, when administered appropriately, bestow benefits upon the host [10]. In addition to their positive impact on intestinal health [8], probiotics have been extensively investigated as a potential therapeutic intervention for metabolic diseases, with the potential to reduce inflammatory mediators [11,12]. It is demonstrated that the administration of the probiotic *Lactobacillus casei* CRL 431 for a duration of two months resulted in the reduction of proinflammatory cytokines, such as interferon-γ, tumor necrosis factor-α (TNF-α), interleukin (IL)-6, and IL-17, in the small intestine and liver of diet-induced obese mice [13]. Furthermore, it was observed that another probiotic, *L. plantarum* LP104, when administered for eight weeks, effectively prevented hyperlipidemia caused by an HFD [14]. The authors also demonstrated the activation of the antioxidant pathway, specifically via nuclear factor E2-related factor 2 (Nrf2), resulting in increased Nrf2 expression in the liver of animals fed an HFD supplemented with probiotics. The administration of *E. faecium* and *L. rhamnosus* probiotics for a period of 28 days not only reduced oxidative markers in the cortex, hippocampus, and striatum, but also enhanced the activity of antioxidant enzymes in these regions, accompanied by an increase in dopamine levels [11].

Furthermore, probiotics have been recognized as a potential treatment for cognitive impairment [15,16]. In a review study, the effectiveness of specific probiotic substances in reducing anxiety-like behaviors in animal models was reported [17]. In another study, regulatory effects of *Lactobacillus plantarum*-derived extracellular vesicles on neuronal function and stress-induced depressive-like behavior were demonstrated [18]. The study indicated the antidepressant effects of probiotics, which reversed the reduced expression of genes related to neurotrophic factors in the hippocampus.

Therefore, a probiotic candidate, *Lacticaseibacillus rhamnosus* LB1.5, derived from raw buffalo milk, was selected due to its effects and probiotic potential, as evidenced by its maintained cell viability following in vitro gastrointestinal simulation [19]. However, the effects of this probiotic on behavior, systemic inflammatory profile, and gene expression in the cortex remain unknown. Consequently, this study aims to evaluate the effect of the *Lact. rhamnosus* LB1.5 on anxious-like behavior, gene expression, and neuroinflammation in the cerebral cortex and hippocampus of adult males fed a HFD.

## 2. Materials and Methods

### 2.1. Animals

Male (*n* = 40) isogenic mice (*Mus musculus*) of the C57BL/6 strain, aged 21 days old, with an average weight of 11.5 g were obtained from the Animal Housing Facility of Universidade Federal de Ciências da Saúde de Porto Alegre (UFCSPA). The animals were housed and maintained in acrylic enclosures with controlled conditions, including a temperature of 23 ± 1 °C, humidity at 55 ± 5%, and a 12 h light/dark cycle with lights turning on at 7 a.m. The Ethics Committee for the Use of Animals at UFCSPA approved this study (no. 722/21), and all procedures followed the ethical rules established by the International Guiding Principles for Biomedical Research Involving Animals (Council for International Organizations of Medical Sciences—CIOMS), Guidelines for Animal Care and Use of Laboratory Animals of the National Institutes of Health, and the Procedures for the Scientific Use of Animals Act (Act # 11.794, 8 October 2008).

### 2.2. Treatments

At 21 days old, shortly after weaning, males were randomly and allocated into the following groups: (i) control (CONT), (ii) control plus probiotic (CONT + PROB), (iii) high-fat diet (HFD), and (iv) high-fat diet plus probiotic (HFD + PROB). The CONT and CONT + PROB groups were given with standard chow (Nuvital, Curitiba, Brazil), with an energy content of 3.4 kcal/g, while the HFD and HFD + PROB groups were given chow (Pragsoluções Biociências, Jaú, Brazil) with an energy content of 5.0 kcal/gplus standard chow, with the aim of replacing micronutrients and tooth wear. Food and water were offered ad libitum. The composition of diets was described previously (see [20]).

The diets (CONT and HFD) were administered daily for 13 weeks. Simultaneously, *Lact. rhamnosus* LB1.5 (a probiotic candidate, derived from raw buffalo milk) was supplemented, at a dose of 3.1 × 10^8^ CFU/mL [19,20], by gavage in the light cycle by 3 times per week. Groups without probiotic supplementation were administered skim milk via gavage, at the same conditions to the probiotic groups (volume and frequency). During this period, food intake was measured weekly, and the data were used to calculate the average daily intake. Weight gain evolution was evaluated by weight measured weekly. At the end of the experiment, the body mass index (BMI) specific to rodents was estimated by the ratio between the weight and length (nose-anus) to evaluate obesity [21].

### 2.3. Light–Dark Box Test

Around 105 days of age, the light–dark box (LDB) task was used to assess anxiety-like behavior [22]. The apparatus consists of an open field with a light background (200 mm in height × 265 mm in width and 260 mm in length) with an opening in one of the walls that connects it to a dark compartment at the bottom, with walls, and cover (200 mm in height × 265 mm wide and 175 mm long). In this test, the animal was placed in the center of the light part of the apparatus, facing the opposite wall of the communication opening of the environment, and its behavior was evaluated for 5 min [23,24]. The tests were recorded and evaluated by the ANY-maze^®^ 7.2 software (Stoelting Co., Wood Dale, IL, USA).

### 2.4. Tissue and Blood Collection

At 110 days of age, in the morning, the animals received, via intraperitoneal administration, a dose of ketamine (240 mg/kg) and xylazine (30 mg/kg) and, afterwards, were euthanized by decapitation. Trunk blood was collected in sterile tubes, centrifuged at 3000 rpm for 10 min at 4 °C, then separated, and stored at −80 °C for biochemical analysis. The prefrontal cortex of the right hemisphere was macroscopically dissected and preserved at −80 °C for subsequent molecular analysis. The left hemisphere was separated and fixed in a zinc buffer solution for later histological analysis.

### 2.5. Cytokines Assay

Utilizing the Luminex xMAP technology, a multiplexed immunoassay was conducted employing Rat Cytokine/Chemokine Magnetic Bead Panels. This assay aimed to quantify interleukin-1α (IL-1α), interleukin-1β (IL-1β), interleukin-6 (IL-6), and interleukin-10 (IL-10) levels in serum. The experimental procedures followed the guidelines provided by the manufacturer.

### 2.6. Histological Analysis and Quantification of Cell

After collection, the left hemisphere underwent fixation in a zinc buffer solution (pH 7.4) for 48 h at room temperature. Subsequently, it underwent histological processing with ethanol/xylene overnight and was embedded in paraffin. The paraffin-embedded tissue was then sectioned (5 μm thick) using a microtome. Following sectioning, the tissue sections underwent treatment with 3% H_2_O_2_ in 10% MeOH for 30 min, followed by a PBS wash for 30 min. Subsequently, the sections were incubated for 30 min in 3% goat serum (Millipore, Burlington, MA, USA) in PBS containing 0.4% Triton X-100 (PBS-T). Further, the sections were subjected to an overnight incubation at 4 °C with primary antibodies (anti-GFAP, 1:750, Millipore, Burlington, MA, USA; anti-Th, 1:50, Bioss, Woburn, MA, USA; IBA1, 1:400, Invitrogen-ThermoFisher Scientific, Waltham, MA, USA).

The immunoreaction was initiated by utilizing a mixture containing 0.06% DAB (3,3′-diaminobenzidine tetrahydrochloride; Sigma-Aldrich, St. Louis, MO, USA) and 0.005% H_2_O_2_ in PBS. Subsequently, tissue sections underwent incubation with a secondary antibody, specifically anti-mouse IgG peroxidase-conjugated (Sigma-Aldrich), at a dilution of 1:500 for 90 min at room temperature. Following this, sections were subjected to counterstaining with hematoxylin, underwent dehydration, and were sealed with Entellan (Merck, Rahway, NJ, USA) along with coverslips.

Five animals per group were analyzed. In the analyzes of the cerebral cortex (GFAP, IBA and Th analysis), three randomly selected fields in non-adjacent sections were selected. In the hippocampus (GFAP and IBA analysis), two fields were randomly selected in each region of the hippocampus, CA1 and Dentate Gyrus. Astrocytes and microglia counts and morphological analysis of digital images were acquired with the EVOS FL auto 2 equipment, using a 40× objective. All GFAP+, IBA and Th cells in the fields were counted [25]. Cell count per field was presented in the results, with ImageJ software utilized for image analysis (National Institutes of Health, Bethesda, MD, USA).

### 2.7. Molecular Analysis

#### 2.7.1. Total RNA Extraction

The TRIzol^®^ method (Invitrogen, São Paulo, Brazil) was used for RNA extraction from the prefrontal cortex, according to manufacturer specifications. Subsequent steps involved chloroform incubation, centrifugation, and extraction of the aqueous phase for RNA isolation. Quantification was determined by the BioSpec-nano^®^ spectrometer (Shimazu, Kyoto, Japan) at 260 nm and 280 nm.

#### 2.7.2. RT-PCR

The cDNA synthesis, following total RNA extraction, was performed using the GoScript™ Reverse Transcription Kit by Promega (São Paulo, Brazil). The process involved incubating 1000 ng of RNA with oligo (dT), dNTPs, and DEPC water, followed by additional steps, including buffer solution, DTT, RNaseOUT, and the M-MLV-RT enzyme, with specific incubation conditions.

#### 2.7.3. Analysis of Gene Expression by qPCR

Investigating transcriptional-level gene expression in the frontal cortex, we evaluated sirtuin (Sirt1), nuclear factor E2-related factor 2 (Nrf2), and brain-derived neurotrophic factor (Bdnf) using specific cDNA amplification primers (10 ng per well). The internal control housekeeping gene, Actin-beta (Actb) [26], was utilized. The primers, acquired from Invitrogen in São Paulo, Brazil, possessed the following sequences: β-Actin—F: 5′AGATCAAGATCATTGCTCCTCCT′3 and R: 5′ACGCAGCTCAGTAACAGTCC′3; Sirt1—F: 5′GGCCGCGGATAGGTCCATA′3 and R: 5′ACAATCTGCCACAGCGTCAT′3; Nrf2—F: 5′GCCCACATTCCCAAACAAGAT′3 and R: 5′CCAGAGAGCTATTGAGGGACTG′3; Bdnf—F: 5′TTGTTTTGTGCCGTTTACCA’3 and R: 5′GGTAAGAGAGCCAGCCACTG′3. Analysis of the amplification products was conducted using the SYBR™ Green method through Real-Time PCR (Life Technologies, Sao Paulo, Brazil). The cycle threshold (CT) value of each reaction determined the mRNA expression level, normalized to the reference gene analyzed simultaneously on the same reaction plate. Relative quantification, representing the mean of normalized expression to the housekeeping gene, was calculated using the 2^−ΔCT^ method [27].

### 2.8. Statistical Analyses

Statistical analyses were conducted utilizing GraphPad Prism 10 from La Jolla, CA, USA. The obtained results underwent evaluation through two-way analysis of variance (ANOVA) followed by Bonferroni’s multiple comparison test. ANOVA considered treatment (vehicle or probiotic) and diet (standard chow or HFD) as principal factors. Data were presented as mean ± S.E.M, and statistical significance was determined at *p* < 0.05.

## 3. Results

### 3.1. Weight Gain, Food Intake, BMI, and Biochemical Parameters

After 13 weeks of intervention, an increase in weight gain (diet effect: F(1,30) = 8.424, *p* = 0.0069), food intake per week (diet effect: F(1,30) = 8.953, *p* = 0.0055) and BMI (diet effect: F(1,27) = 8.186, *p* = 0.0081) was observed in the HFD group compared to the CONT group. On the other hand, no effect of probiotic supplementation on weight gain, food intake per week, and BMI was observed (Table 1). Furthermore, HFD increased serum levels of total cholesterol (*p* < 0.0001), HDL (*p* = 0.0125) and LDL (*p* = 0.0149) compared to CONT. In addition, no difference in serum glucose and triglycerides was observed between the diets or among the groups that received the probiotic (*p* > 0.05).

### 3.2. Inflammatory Cytokines

**Figure 1:**
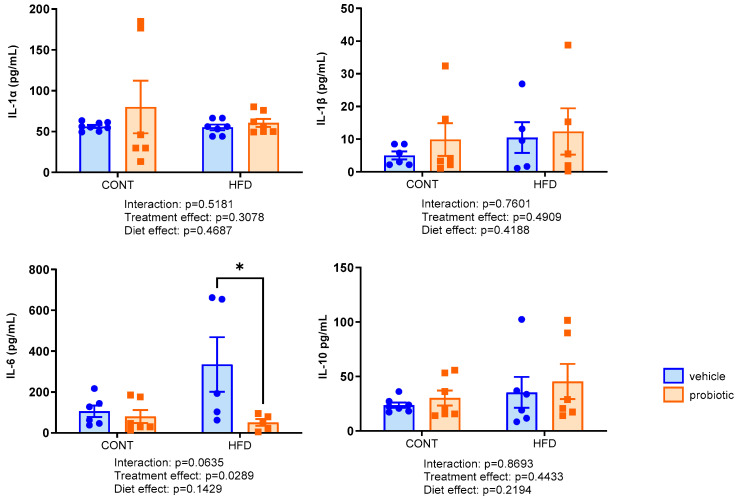
Analysis of serum levels of cytokines of the adult males fed with standard (CONT, *n* = 6–8), standard plus probiotic (CONT + PROB, *n* = 6–7), high-fat (HFD, *n* = 5–7) and high-fat plus probiotic (HFD + PROB, *n* = 4–7) diets for 13 weeks by two-way ANOVA followed by Bonferroni’s multiple comparison test. Results, presented as mean ± SEM, indicated significant differences (* *p* < 0.05) compared to the high-fat diet group (HFD). Abbreviations: interleukin 6 (IL-6), interleukin 1α (IL-1α), interleukin 1β (IL-1β) and interleukin 10 (IL-10).

The systemic inflammatory status was evaluated by measuring serum levels of IL-1α, IL-1β, IL-6, and IL-10 (Figure 1). We observed a significant effect of probiotic supplementation only on the concentration of IL-6 (F(1,18) = 6.636, *p* = 0.0289). The HFD + PROB group exhibited decreased IL-6 levels compared to the HFD group (*p* = 0.03). The other cytokines evaluated did not show significant differences between the groups (*p* > 0.05).

### 3.3. Anxious-like Behavior

Table 2 presents the results of the LDB test. Regarding the latency of the first transition from the light to the dark compartment, we observed a longer latency in animals treated with the probiotic (*p* = 0.0079). In addition, probiotic supplementation (CONT and HDF groups) increased the total distance traveled in the light compartment (*p* = 0.0489) and remained longer in the light compartment (*p* = 0.0466) than their HFD and CONT groups.

### 3.4. Neuroinflammation Markers in the Cerebral Cortex and Hippocampus

**Figure 2:**
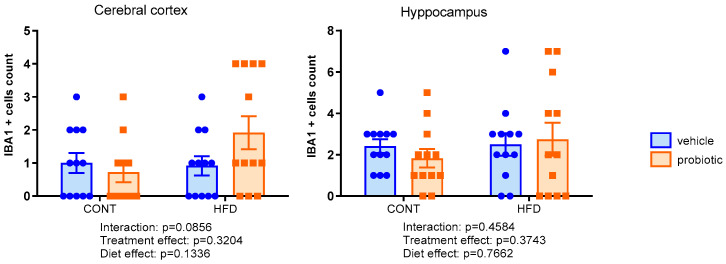
Number of IBA1 positive cells in the cerebral cortex and hippocampus (CA1 area and dentate gyrus) of the adult males fed with standard (CONT), standard plus probiotic (CONT + PROB), high-fat (HFD) and high-fat plus probiotic (HFD + PROB) diets for 13 weeks by two-way ANOVA followed by Bonferroni’s multiple comparison test. Results were presented as mean ± SEM, with  5 animals per/group.

**Figure 3:**
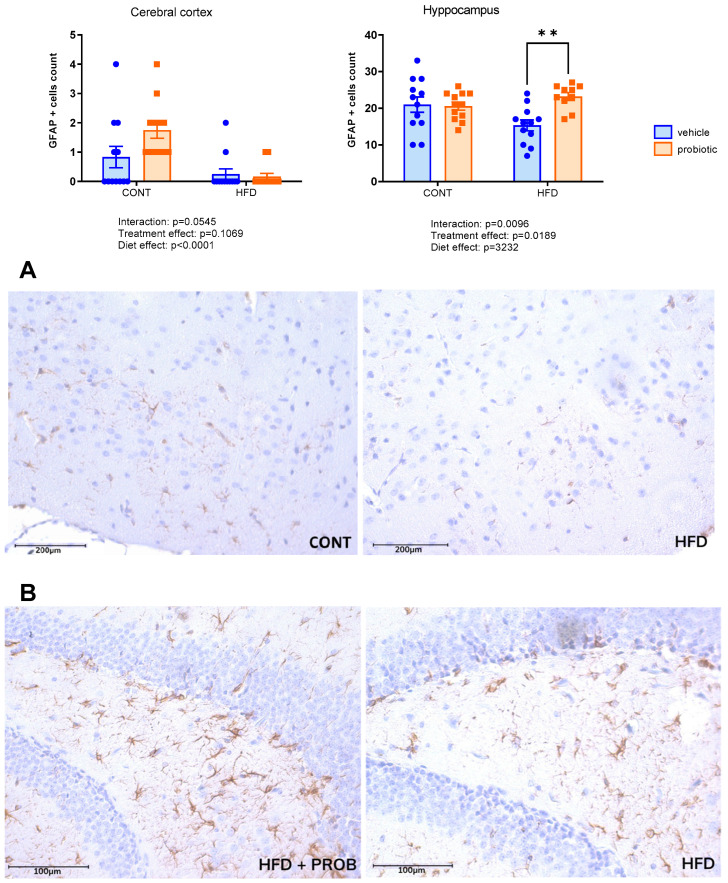
Number of GFAP positive cells in the cerebral cortex and hippocampus (CA1 area and dentate gyrus) of the adult males fed with standard (CONT), standard plus probiotic (CONT + PROB), high-fat (HFD) and high-fat plus probiotic (HFD + PROB) diets for 13 weeks by two-way ANOVA followed by Bonferroni’s multiple comparison test. (**A**) Representative images of GFAP positive cells in the cerebral cortex immunofluorescent under confocal microscopy (bar = 200 μm). (**B**) Representative images of GFAP positive cells in the cerebral hippocampus immunofluorescent under confocal microscopy (bar = 100 μm). Results, presented as mean ± SEM, indicated significant differences (** *p* < 0.01) compared to the high-fat diet group (HFD). Scale bar = 100 µm and 200 µm, cerebral cortex and hippocampus, respectively. *n* = 5/group.

Figure 2 shows the IBA1 immunoreactivity in the cerebral cortex and hippocampus. There were no differences among the groups in both regions evaluated, regardless of the diet and/or probiotic supplementation. In relation to GFAP-positive cells in the cerebral cortex (Figure 3), we observed a significant interaction between diet and probiotics supplementation (F(1,44) = 3.901, *p* = 0.0545). In addition, in HFD-fed animals, there was a decreased number of GFAP-positive cells compared to CONT groups (diet effect: F(1,44) = 18.32, *p* < 0.0001). In the hippocampus, we identified an interaction between diet and supplementation (F(1,42) = 7.370, *p* = 0.0096). We also observed a probiotic supplementation effect (F(1,42) = 4.361, *p* = 0.0189) in relation to GFAP immunoreactivity (Figure 3). The post hoc test showed that the HFD + PROB group had an increased number of GFAP-positive cells compared to the HFD group (*p* = 0.001).

**Figure 4:**
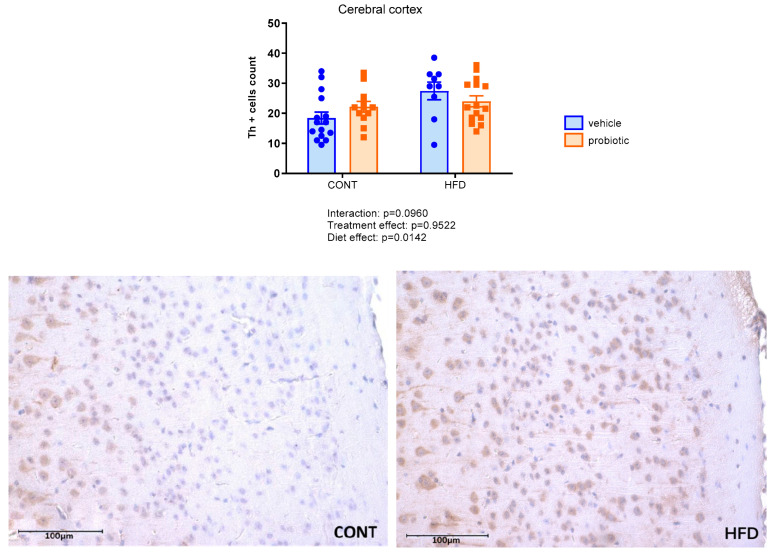
Number of Th positive cells in the cerebral cortex of the adult males fed with standard (CONT), standard plus probiotic (CONT + PROB), high-fat (HFD) and high-fat plus probiotic (HFD + PROB) diets for 13 weeks by Bonferroni’s multiple comparison test. Results were presented as mean ± SEM. Scale bar = 100 µm. *n* = 5/group.

Figure 4 shows a higher number of Th-positive cells in the cerebral cortex in HFD-fed mice (diet effect: F(1.47 = 6.487, *p* = 0.0142). However, there was no difference in the effect of probiotic supplementation on Th immunoreactivity.

### 3.5. Gene Expression of Sirt1, Nrf2 and Bdnf in the Prefrontal Cortex

**Figure 5:**
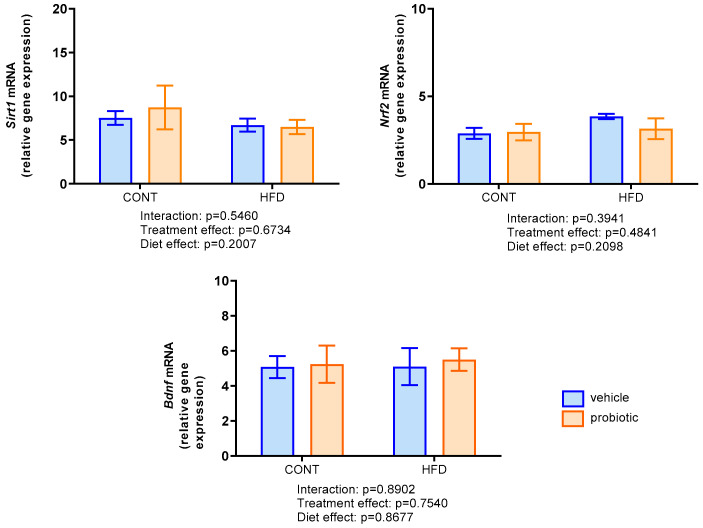
Relative gene expression of Sirt1, Nrf2 and BDNF in the prefrontal cortex of the adult males fed with standard (CONT, *n* = 6–8), standard plus probiotic (CONT + PROB, *n* = 6–7), high-fat (HFD, *n* = 5–7) and high-fat plus probiotic (HFD + PROB, *n* = 4–7) diets for 13 weeks by two-way ANOVA followed by Bonferroni’s multiple comparison test. Results were presented as mean ± SEM.

*Sirt1*, *Nrf2* and *Bdnf* relative gene expression in the prefrontal cortex was evaluated by qPCR analysis (Figure 5). There were no differences among the studied groups in *Sirt1*, *Nrf2*, and *Bdnf* gene expression (*p* > 0.05).

## 4. Discussion

In the present study, we showed that the consumption of HFD after weaning, over 13 weeks, led to an increase in body weight and serum lipid concentrations. The HFD also had an impact on GFAP and Th immunoreactivity in the cerebral cortex. In the hippocampus, we observed an interaction between diet and supplementation, where the HFD + PROB group exhibited an increased number of GFAP-positive cells compared to the HFD group. Additionally, supplementation with the probiotic *Lact. rhamnosus* LB1.5 resulted in a decrease in serum IL-6 levels in HFD-fed mice and promoted a reduction of anxiety-like behavior, as assessed in the LDB test.

Previous evaluations in vitro detailed that *Lactobacillus rhamnosus* LB1.5 possesses the ability to adhere and aggregate with the intestinal epithelium, exhibits tolerance to the acidic environment, and resists bile salts [19]. Thus, the probiotic, derived from raw buffalo milk, has been shown to be a promising candidate for human health. In the testing conducted in this experiment, the probiotic candidate was well-tolerated by the gastrointestinal tract of all animals, as they remained healthy throughout the experiment.

Despite the impact of HFD on obesity and associated metabolic disorders being well-documented in the literature [28,29,30], the effects of probiotic *Lact. rhamnosus* LB1.5 supplementation on these parameters remains relatively understudied. In the present study, probiotic supplementation, at the administered dose and frequency, did not influence metabolic alterations induced by HFD (weight gain, BMI, and cholesterol and LDL levels increased). On the other hand, studies have shown that other probiotics, such as *Lact. rhamnosus* 86 (isolated from Korean infant feces), at a higher dose (10^10^ CFU/day) during the same period (12 weeks), presented an anti-obesity effect, and reduced cholesterol levels in 10-week-old male C57BL/6 J mice [31]. It has also been shown that the other strain supplementation (*Lact. rhamnosus* LRH05, at 10^9^ CFU/day for 10 weeks) significantly decreased body weight gain and serum TG levels in 7-week-old male C57BL/6 mice [12]. The lack of observed anthropometric and metabolic effects of probiotic supplementation in our study may be related to some factors. Firstly, in our study, we chose to assess the effect of the probiotic soon after weaning and opted for a lower weekly frequency (3 times/week), a regimen extensively employed in animal studies [32,33,34,35,36]. Moreover, in this study, we used a probiotic produced from buffalo milk which, under the tested conditions, may not have exhibited anti-obesogenic effects. These decisions stem from the novelty of our investigation, marking the first assessment of the impacts of *Lact. rhamnosus* LB1.5 supplementation soon after weaning.

It has also been shown that proinflammatory cytokines are altered by the use of HFD, including plasma interleukins such as IL-6, IL-1β, IL-6, IL-18, and TNF-α in mice [31,37,38,39]. In this context, a cytokine of particular interest is IL-6, which plays a pivotal role in the body’s inflammatory processes and has been implicated in various health conditions, including metabolic disorders and diseases associated with inflammation [37,40]. Evaluating the *L. casei* CRL 431 supplementation (4 ± 2 × 10^8^ CFU/mL, for two months) in HFD mice, Núñez and collaborators [13] verified that the probiotic used induced an anti-inflammatory response, resulting in the decrease of proinflammatory cytokines (IL-6, IL-17, and TNF-α) in the small intestine, liver, and adipocytes. In contrast, we observed that among all the evaluated cytokines, only the serum IL-6 levels were elevated in animals treated with HFD, and *Lact rhamnosus* LB1.5 supplementation was able to reduce this cytokine in serum. This finding may serve as an indicator of the potential anti-inflammatory properties of the probiotic utilized in this study, however, more studies are needed to investigate the mechanism of action of the IL-6 cytokine associated with the probiotic used.

Additionally, chronic inflammation also characterizes the state of overweight or obesity, and the HFD consumption activates inflammatory pathways in peripheral tissues. This activation may potentially extend to the brain, affecting regions such as the hypothalamus, hippocampus, and cortex [37,41,42,43], and consequently induces behavior alterations [44,45]. In rodent models, it has been revealed that there is an increased anxiety-like behavior and symptoms resembling depression in response to HFD [17,46,47,48]. This has been evidenced by reduced time spent in the central zone and a decrease time spent on the light side in the LDB test [48]. In our present study, mice supplemented with *Lact. rhamnosus* LB1.5 showed reduced anxiety-like behavior, as evidenced by their increased time spent in the light compartment, regardless of their dietary group (CONT or HFD). These results are in line with Han and collaborators’ systematic review, suggesting that probiotics may contribute to anxiety reduction, with a specific focus on the probiotic species *Lact. rhamnosus* [48]. Thus, these findings open up a promising avenue for further investigations into *Lact. rhamnosus* LB1.5 and its relationship with gut microbiota, inflammation, and behavioral outcomes.

Inflammation induced by HFD consumption, coupled with hypothalamic dysfunction resulting from excessive exposure to fatty acids [49,50,51], has the potential to extend its deleterious effects to various brain regions, including the hippocampus and cerebral cortex [41]. Prolonged adherence to an HFD regimen can lead to neuroinflammation within the hippocampus due to increased permeability of the blood–brain barrier, subsequently activating astrocytes and microglia in response to disruptions in brain homeostasis [43,51]. Our study showed a reduction in the number of GFAP-positive cells within the cerebral cortex of animals subjected to an HFD, without effect of probiotic. In the hippocampus, we observed an interaction between dietary composition and probiotic supplementation, with this group displaying an elevated count of GFAP-positive cells compared to the HFD group, suggesting the occurrence of reactive astrogliosis.

It is well-established that obesity induces brain atrophy in both humans and animal models, a phenomenon likely associated with disturbances in cerebral vascular function and concurrent inflammation [52]. Nevertheless, the precise underlying mechanisms remain incompletely elucidated [53,54,55]. An alternative approach for assessing neuroinflammation within neural tissue involves the examination of microglial activation [56]. The IBA1 protein is expressed in microglia, crucial components in the defense of the nervous system and participants in brain defense mechanisms, as well as various pathological conditions [57]. In our investigation, we evaluated IBA1 immunoreactivity within the cerebral cortex and hippocampus across different experimental groups. Unlike the findings of Saiyasit et al. [58], who documented a substantial increase in the number of IBA-1 positive cells following a 12-week administration of HFD in mice, our study did not observe immunoreactivity for IBA1 within the examined brain regions, either as a result of HFD consumption or probiotic supplementation. In another study, besides confirming alterations in microglial morphology (including an increase in Iba-1-positive cells) caused by HFD, the authors also verified that supplementation with *Lact. paracasei* (1 × 10^8^ cfu, 1 mL/day for 12 weeks) mitigated these effects [59].

Research indicates a relationship between inflammatory processes and the dopaminergic system within the brain [60]. In this regard, Th has been shown to play a crucial role in neuronal function and the regulation of food intake through the dopaminergic system [61]. Moreover, both high and low-calorie diets exert influence over the activity and gene expression associated with the dopaminergic system across diverse brain regions, notably the mesolimbic pathway originating in the ventral tegmental area (VTA) and projecting to structures such as the nucleus accumbens, amygdala, hippocampus, and prefrontal cortex [62]. Our results indicated that HFD increased the number of Th-positive cells only in the cerebral cortex, with no observable changes attributed to the probiotic intervention. It is posited that changes in Th expression primarily stem from macronutrient intake rather than fluctuations in body weight. Prior research further substantiates the HFD-induced upregulation of Th gene expression, emphasizing the complex interplay among diet, dopamine synthesis, inflammation and neural function across a spectrum of brain regions [63,64].

The consumption of a HFD may lead to obesity, initiating an inflammatory state and subsequently promoting oxidative stress [4,65,66], which can also lead to neuroinflammation [67]. Within the central nervous system, including the prefrontal cortex, safeguarding mechanisms against oxidative stress play a fundamental role in protection against neuroinflammation. Proteins like Sirt1 and Nrf2 assume crucial roles in regulating metabolism and responding to oxidative challenges, particularly with HFD consumption [68,69]. Sirt1 has emerged as a protective factor against the adverse metabolic effects of HFD, modulating various cellular processes, including energy metabolism and stress response [70]. These beneficial effects, stemming from multifaceted mechanisms, inducing antioxidant proteins and attenuation of the activation of proinflammatory cytokines, such as TNFα and IL-6 [71]. On the other hand, HFD has been demonstrated to impact the Nrf2 pathway, adversely affecting essential cellular processes crucial for brain health. The modulation of Nrf2 by HFD not only inhibits its activity, but also exacerbates oxidative stress, leading to alterations in the release of inflammatory mediators. This dual impact on Nrf2 has significant implications for overall brain health and homeostasis [72]. Contrary to these findings, our results indicate that the gene expression levels of Sirt1 and Nrf2 in the prefrontal cortex remain unchanged, exhibiting no fluctuations in response to either HFD or probiotic supplementation.

Furthermore, we explore the role of Bdnf in neuroprotection and synaptic plasticity. Studies have linked HFD to altered Bdnf levels, including in the prefrontal cortex, and their potential impact on learning and memory [73]. In contrast, our findings indicated that neither the HFD nor probiotic supplementation influenced Bdnf gene expression in the prefrontal cortex of male mice. This suggests a region-specific response, highlighting the stability of Bdnf expression in our experimental setup, unaffected by the HFD diet with 13 weeks’ duration and 57.2% fat content or probiotic supplementation. Additionally, compensatory adaptations play a pivotal role in mitigating HFD’s detrimental effects on Bdnf gene expression. These adaptations encompass alternative signaling pathways, positive regulation of protective factors affecting the Bdnf gene, and epigenetic modifications, such as DNA methylation and histone modifications. Together, these mechanisms maintain sufficient Bdnf levels despite challenging dietary conditions. The crosstalk between Bdnf and other neurotrophic factors, potentially influenced by probiotics, amplifies the neuroprotective response. This complex interplay underscores the body’s resilience in preserving Bdnf expression despite adverse dietary challenges [74,75,76].

Although our study represents the first evaluation of the biological effects of the candidate probiotic *Lact. rhamnosus* LB1.5, we acknowledge certain study limitations. Firstly, we utilized C57BL/6 mice, a strain known for obesity susceptibility on a high-fat diet (HFD). However, obesity induction can also be influenced by strain variations, posing an additional challenge [77]. Unfortunately, we could not analyze the genetic profile of the mice, presenting a notable limitation to our study. Secondly, our approach involved evaluating the effect of the probiotic soon after weaning at a weekly frequency of 3 times/week, possibly limiting the anti-obesity impact. Thirdly, in contrast to other studies starting diets with older animals [12,31], our study initiated the diet and probiotics soon after weaning, possibly influencing the observed effects. Therefore, future investigations should explore the probiotic’s effects in females, with higher probiotic frequency administration, investigating other candidate genes in different areas of the CNS.

## 5. Conclusions

In summary, our 13-week intervention showed that probiotic *Lact. rhamnosus* LB1.5 supplementation, administered at a dose of 1.3 × 10^8^ CFU/mL and obtained from buffalo milk, led to a decrease of IL-6 concentration in HFD-fed mice. Furthermore, it promoted a reduction in anxiety-like behavior. The HFD had an impact on GFAP and Th immunoreactivity in the cerebral cortex. In the hippocampus, we observed an interaction between diet and supplementation, with HFD + PROB showing an increased number of GFAP-positive cells compared to HFD. This demonstrates that nutrition in the early stages plays an important role in the development of health and disease in adulthood. Future studies are required to elucidate the mechanisms through which *Lact. rhamnosus* LB1.5 supplementation promotes anxiolytic effects in obesity. However, it can be assumed that *Lact. rhamnosus* LB1.5 supplementation may represent a potential target for therapeutic intervention.

## Figures and Tables

**Table 1:** Effect of consumption of standard diet (CONT), standard diet + probiotics (CONT + PROB), high-fat diet (HFD), and high-fat diet + probiotics (HFD + PROB) in male mice on biochemical parameters, body mass index and food intake per week.

Parameters	CONT (*n* = 10)	CONT + PROBIOTIC (*n* = 10)	HFD (*n* = 10)	HFD + PROBIOTIC (*n* = 10)	Two-Way ANOVA		
					Interaction	Diet Effect	Treatment Effect
**Glucose mg/dL)**	179.34 ± 15.06	173.52 ± 10.61	193.84 ± 14.68	194.01 ± 9.02	0.8114	0.1695	0.8220
**Triglycerides (mg/dL)**	84.75 ± 12.84	107.02 ± 14.00	76.72 ± 7.50	91.56 ± 10.81	0.7557	0.3286	0.1269
**Total cholesterol (mg/dL)**	58.34 ± 5.41	55.34 ± 5.708	105.85 ± 7.83	89.77 ± 8.68	0.3596	<0.0001	0.1845
**HDL ^a^ (mg/dL)**	24.43 ± 2.87	30.16 ± 3.14	46.26 ± 7.88	34.89 ± 4.96	0.0962	0.0125	0.5736
**LDL ^b^ (mg/dL)**	27.36 ± 9.04	23.83 ± 8.83	63.83 ± 13.83	45.13 ± 9.26	0.5008	0.0149	0.3263
**Body Mass Index (g/cm^2^)**	0.52 ± 0.02	0.52 ± 0.02	0.63 ± 0.04	0.59 ± 0.04	0.5182	0.0081	0.5908
**Food intake per week (g)**	11.83 ± 0.99	10.39 ± 1.04	13.57 ± 0.76	14.64 ± 1.10	0.2203	0.0055	0.8504

^a^ HDL: high-density lipoprotein; ^b^ LDL low-density lipoprotein. Results were presented as mean ± SEM. Two-way analysis of variance was conducted, followed by the Bonferroni multiple comparison test.

**Table 2:** Effect of consumption of standard diet (CONT), standard diet + probiotics (CONT + PROB), high-fat diet (HFD), and high-fat diet + probiotics (HFD + PROB) in male mice submitted to the light/dark test for 5 min.

Behaviors	CONT (*n* = 10)	CONT + PROBIOTIC (*n* = 10)	HFD (*n* = 10)	HFD + PROBIOTIC (*n* = 10)	Two-Way ANOVA		
					Interaction	Diet Effect	Treatment Effect
**Latency to the first transition (s)**	17.49 ± 4.75	29.63 ± 5.28	8.19 ± 4.60	26.48 ± 6.60	0.5696	0.2527	0.0079
**Time spent in the light compartment (s)**	147.40 ± 21.27	184.78 ± 12.24	149.34 ± 20.75	192.11 ± 20.46	0.8911	0.8141	0.0489
**Total distance traveled in the light compartment (m)**	4.38 ± 0.63	5.27 ± 0.56	4.01 ± 0.66	5.39 ± 0.20	0.6812	0.8510	0.0466

Results were presented as mean ± SEM. Two-way analysis of variance was conducted, followed by the Bonferroni multiple comparison test. s: seconds; m = meters.

## Data Availability

The data that support the findings of this study are available on reasonable request from the corresponding author.
